# The Non-Canonical Aspects of MicroRNAs: Many Roads to Gene Regulation

**DOI:** 10.3390/cells8111465

**Published:** 2019-11-19

**Authors:** Christiaan J. Stavast, Stefan J. Erkeland

**Affiliations:** Department of Immunology, Erasmus University Medical Center, Dr. Molenwaterplein 40, 3015GD Rotterdam, The Netherlands; c.stavast@erasmusmc.nl

**Keywords:** MicroRNAs, biogenesis, nuclear localization, transcriptional regulation, non-canonical

## Abstract

MicroRNAs (miRNAs) are critical regulators of gene expression. As miRNAs are frequently deregulated in many human diseases, including cancer and immunological disorders, it is important to understand their biological functions. Typically, miRNA-encoding genes are transcribed by RNA Polymerase II and generate primary transcripts that are processed by RNase III-endonucleases DROSHA and DICER into small RNAs of approximately 21 nucleotides. All miRNAs are loaded into Argonaute proteins in the RNA-induced silencing complex (RISC) and act as post-transcriptional regulators by binding to the 3′- untranslated region (UTR) of mRNAs. This seed-dependent miRNA binding inhibits the translation and/or promotes the degradation of mRNA targets. Surprisingly, recent data presents evidence for a target-mediated decay mechanism that controls the level of specific miRNAs. In addition, several non-canonical miRNA-containing genes have been recently described and unexpected functions of miRNAs have been identified. For instance, several miRNAs are located in the nucleus, where they are involved in the transcriptional activation or silencing of target genes. These epigenetic modifiers are recruited by RISC and guided by miRNAs to specific loci in the genome. Here, we will review non-canonical aspects of miRNA biology, including novel regulators of miRNA expression and functions of miRNAs in the nucleus.

## 1. Introduction

### MiRNA Biogenesis and Function

MicroRNAs (miRNAs) are small non-coding RNAs that are involved in gene expression regulation. Thousands of miRNAs have been identified and are recorded in the online database: miRbase (www.mirbase.org), which currently contains 1,917 miRNA entries for the human genome [1]. The biogenesis of miRNAs is a rapid process. Studies in *Drosophila* have shown that the biogenesis of miRNAs ranks as one of the fastest among transcripts: At least 40% of the mature miRNAs are produced within 5 min [2]. Like protein-encoding genes, miRNAs may have independent transcriptional regulatory units but are also frequently located within introns of host genes, suggesting co-regulation of transcription. However, miRNA expression does not necessarily correlate with the levels of their host gene. Many intronic miRNAs have an independent transcriptional start site and are controlled by a different promoter and/or other regulatory sequences compared to their host gene [3]. For example, super-enhancers, which are involved in cell identity, drive both transcription and processing of miRNAs [4]. Interestingly, super-enhancers are also deregulated in human cancer and may, at least in part, explain the aberrant expression of some oncogenic miRNAs.

MiRNA biogenesis starts with post-transcriptional or co-transcriptional processing of primary miRNA transcripts (pri-miRNAs) from the genome [5,6,7,8,9]. Most canonical pri-miRNAs are transcribed by RNA Polymerase II (POL II), contain a 5′-cap and do not necessarily have a poly-A tail [10] (For reviews see: [3,11,12]). In addition, some miRNAs are located within Alu-regions and other repetitive elements and are regulated by POL II [13]. In the nucleus, pri-miRNAs form a hairpin structure and are cleaved into premature miRNAs (pre-miRNAs) by the RNase III enzyme DROSHA, which is in complex with RNA-binding protein (RBP) DiGeorge syndrome chromosome region 8 (DGCR8) [12,14,15,16,17,18]. The hairpin structure and the distance from the single-stranded RNA (ssRNA) basal segments to the dsRNA junction of the pri-miRNA stem is critical for DROSHA cleavage [19,20]. The pre-miRNA is protected from degradation and exported from the nucleus into the cytoplasm by Exportin-5 (XPO-5) in a RAS-related nuclear protein-guanosine-5’-triphosphate-ase (Ran-GTPase) dependent manner [21,22]. After translocation to the cytoplasm, the pre-miRNA is further processed by the RNase III enzyme DICER, which is bound to trans-activation-responsive RNA-binding protein (TRBP) [12,23]. In this protein complex, TRBP binds the pre-miRNA and DICER cleaves the loop of the hairpin, resulting in a miRNA duplex of approximately 22 nucleotides (nt) with a typical 2 nt overhang on the 3′-end [23,24,25]. Many studies have shown that DICER is essential for the processing of the majority of miRNAs in many organisms and cell types [26,27,28,29,30].

The miRNA duplex interacts with components of the RNA-induced silencing complex (RISC) loading complex (RLC) [31,32,33,34,35,36]. The endonucleolytic cleavage activity of Argonaute 2 (AGO2), also called slicer activity, first destabilizes the miRNA duplex by nicking. Next, the miRNA duplex is unwinded by RNA helicases, such as RNA helicases H and P68 [37,38,39]. Other proteins involved in this process are Translin-associated factor X (TRAX), TRANSLIN and heat shock protein 90 (HSP90), which form an endoribonuclease complex called component 3 of promoter of RISC (C3PO) that degrades the passenger strand [34,37,38,39]. The endonucleolytic cleavage activity of AGO2 is absent in AGO1, AGO3, and AGO4. These proteins separate the strands in a cleavage-independent fashion. In this process, mismatches in the miRNA duplex at positions 2–7 are important for the selection of either the 5p-arm or 3p-arm of the miRNA that is loaded in RISC [40,41,42]. Mature miRNAs bound to AGO are four times more stable compared to mRNAs and may accumulate up to half-a-million copies per cell [2]. 

**Figure 1:**
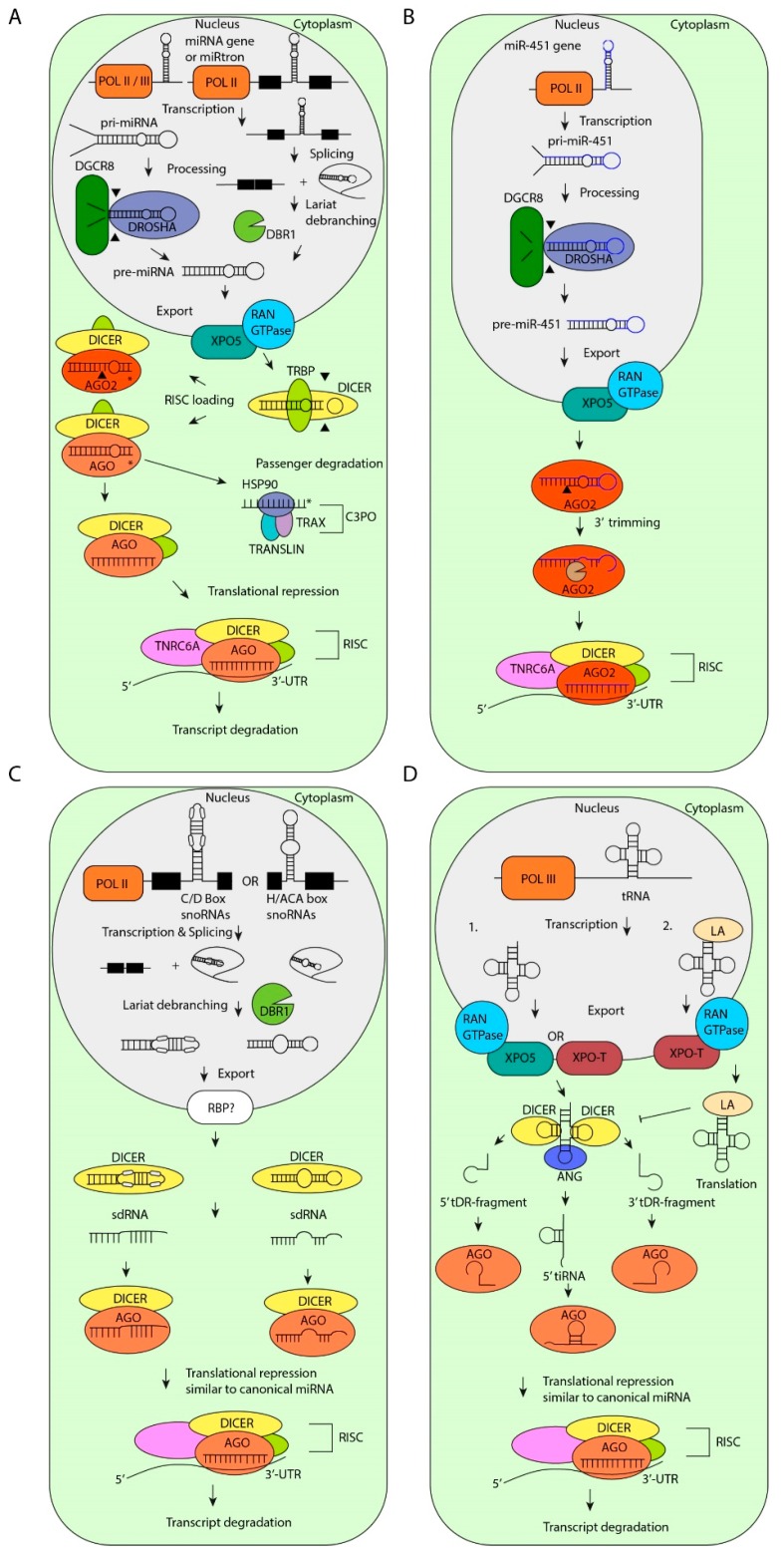
Biogenesis of microRNAs (miRNAs). (**A**) Canonical miRNA biogenesis starts with transcription of miRNA genes by RNA Polymerase II (POL II) or POL III. Next, the primary (pri)-miRNAs are processed by DROSHA/DiGeorge syndrome critical region 8 (DGCR8). The resulting pre-miRNAs are exported to the cytoplasm by Exportin-5 (XPO-5). MiRtrons are spliced out and the intron lariat is debranched by lariat debranching enzyme 1 (DBR1), which results in pre-miRNAs. Once exported, the pre-miRNAs are cleaved by DICER/trans-activation-responsive RNA binding protein (TRBP). Next, the passenger strand is degraded by the component 3 promoter of RNA-induced silencing complex (C3PO) complex. The guide strand, which is loaded into RNA-induced silencing complex (RISC), is involved in translational repression and subsequent transcript degradation. (**B**) MiR-451 is processed in a DICER-independent manner. After processing by DROSHA/DGCR8 and export to the cytoplasm, the passenger strand is degraded by Argonaute 2 (AGO2)-mediated cleavage and trimming. (**C**) Non-canonical processing of small nucleolar RNAs (snoRNAs) results in snoRNA-derived RNAs (sdRNAs). SnoRNAs are spliced from genes and debranched by DBR1. Subsequently, snoRNAs are exported to the cytoplasm by an unknown mechanism and are processed by DICER into sdRNAs which are loaded in RISC. (**D**) Non-canonical processing of transfer RNAs (tRNAs) results in tRNA-derived miRNAs. (1) After transcription, tRNAs are transported to the cytoplasm by XPO-5 or XPO-T. The 5′-loop and the 3′-loop is cleaved by DICER, resulting in 5′-tRNA-derived RNA (tDR)-fragments and 3′-tDR-fragments respectively. The anticodon loop is cleaved by Angiogenin (ANG), resulting in 5′-tRNA stress-induced fragments (tiRNAs). All tDR-fragmentss are subsequently loaded into RISC similar to canonical miRNAs. (2) After transcription, tRNAs can be stabilized by Lupus autoantigen (LA) and exported to the cytoplasm by XPO-T. LA inhibits the processing of tRNAs by DICER and preserves tRNA stability for translation. HSP90: heat shock-Protein 90, TNRC6A: trinucleotide repeat-containing gene 6A.

The miRNA loaded RISC (miRISC) binds to reverse complementary sequences within the 3′-untranslated region (UTR) of target mRNAs [11,43,44]. All AGOs silence their target mRNAs by recruiting downstream factors such as proteins from the Glycine-Tryptophan protein of 182 kDa (GW182) family such as, trinucleotide repeat-containing gene 6A–6C (TNRC6A–TNRC6C) and the carbon catabolite repressor 4–negative on TATA (CCR4-NOT) complex, that mediate translational repression, deadenylation or decapping of target transcripts. [43,45,46,47,48,49] (for a review: [50]) (Figure 1A). In addition, AGO2 silences these targets through its slicer activity. This activity needs a perfect match between the miRNA and the target mRNA in the central region of the miRNA [51]. AGO3 cleaves target RNAs under certain circumstances as well. This activity is highly dependent on the loaded miRNA sequence [52]. AGO1 and AGO4 do not possess slicer activity. The miRNA-mediated RNA silencing processes are mainly localized in processing-bodies (P-bodies) in the cytoplasm [53,54,55,56,57]. 

In general, nucleotides 2–7 at the 5′-end of the miRNA, the so-called seed region, are critical for canonical target binding specificity [11]. Therefore, seed-matching is commonly used for the prediction of miRNA targets. However, the actual interactions of miRNAs with their targets may be cell type-specific and controlled by RNA-binding proteins [58,59]. Further information can be found in the following reviews: [60,61]. The position of miRNA binding to the target mRNA also largely determines the outcome. For instance, miRNAs that interact close to the poly-A tail of mRNAs are more likely to silence their mRNA target [62]. In addition, multiple non-canonical miRNA target recognition sites have been identified: (1) the so-called pivot seed pairing or nucleation bulge, which is common in *C. elegans* and mice, (2) target recognition by center-pairing miRNA-binding sites lacking both perfect seed pairing, and 3’-compensatory pairing, and (3) other more complex pairing modes of the miRNA with the target [63,64,65,66]. However, contradictory studies have provided strong evidence that in mammalian cells some of the identified non-canonical sites do not mediate repression of their bound targets. Importantly, the presented data indicate that the vast majority of functional miRNA-binding sites are canonical [67]. 

Unexpectedly, some miRNAs are destabilized by specific interactions with mRNAs [68,69]. These transcripts contain sequences that have a near-perfect match with miRNAs and contain centered mismatches. This type of interaction causes miRNA-unloading from AGO and destabilization of the 3′-end of the miRNA. This post-transcriptional regulation of miRNAs is also called target-directed miRNA degradation (TDMD) [70]. Recent structural analyses of AGO2 and mutational analyses of miRNAs and their respective targets revealed that the shape of the AGO2 central cleft and the centered mismatches in the miRNA targets allow for modifications of the miRNA 3′-end by unknown enzymes. These modifications lead to 3′-end remodeling and eventually the decay of miRNAs [70]. These findings may at least in part explain the rapid fluctuations of specific intracellular miRNA concentrations during cell development. 

## 2. Non-Canonical miRNAs

Some miRNAs have been identified that are generated by a different biogenesis pathway and are called non-canonical miRNAs. Many non-canonical miRNAs are involved in different human diseases including cancer [71,72,73], as reviewed in [74], whereas others are found to be active in immune cells [74,75,76,77,78]. Below, we focus on recent findings on the mechanistic aspects of non-canonical miRNA biogenesis. 

### 2.1. miRtrons

Recently, alternative miRNA biogenesis pathways have been identified. For example, some non-canonical pri-miRNAs are encoded in introns of coding genes and are named miRtrons [79]. All miRtrons are initially processed by the nuclear splicing machinery like typical introns and form stable hairpins with a shorter stem compared to canonical pri-miRNAs [80]. These shorter hairpin structures cannot be processed by DROSHA/DGCR8, but undergo lariat-debranching by the debranching enzyme 1 (DBR1) instead [81,82]. In full agreement, intron-derived miRNAs persist in cells that are deficient for DROSHA or DGCR8 activity [79,82,83,84]. Like canonical miRNAs, miRtron-derived pre-miRNAs are bound by XPO-5, translocated to the cytoplasm and cleaved by DICER (Figure 1A). Recently, a highly advanced machine learning-based prediction tool has been developed that distinguishes between canonical miRNAs from miRtrons based on hairpin length and GC content [85]. This tool ultimately leads to a better understanding of the characteristics of miRtron processing [85].

### 2.2. Dicer-Independent miRNAs

So far, only one annotated miRNA is known to be processed in a DICER-independent fashion. Pre-miR-451, of which the stem-loop structure is too short to be cleaved by DICER, requires the AGO2 slicer activity for miRNA maturation [86,87]. The length of the stem-loop structure, the imperfect base pairing in the stem and low GC content in the distal stem determine the AGO2-mediated processing of pre-miR-451 and subsequent RISC loading [88] (Figure 1B). In addition, the activity of eukaryotic translation initiation factor 1A (EIF1A), another RISC component, is crucial for miR-451 processing by AGO2 [89]. This knowledge of the properties of the pre-miR-451 has led to the development of better RNAi tools. For example, DICER-independent processing of small hairpin RNAs (shRNAs) have various advantages, including preferential loading of small interfering RNAs (siRNAs) in AGO2, that allows for enhanced RNAi and the opportunity to target genes in DICER-mutant tumor cells (review: [90]). 

### 2.3. snoRNA-Derived miRNAs

Small nucleolar RNAs (snoRNAs) belong to a class of non-coding RNAs with a size of 60–300 nt which is subdivided in H/ACA box snoRNAs (snoRAs, ACAs) and C/D box snoRNAs (SNORDs) and Cajal-specific snoRNAs (sca-RNAs) [91,92]. Recently published data show that snoRNAs control gene expression in different ways, including the regulation of the ribosome by modifying ribosomal RNA (rRNA). The best-understood functions of snoRNAs are the directing of the 2′-*O*-methyltransferase Fibrillarin to specific rRNA sites and the guiding of Dyskerin to rRNAs which pseudouridylates RNA substrates [92,93]. There is increasing evidence that some snoRNAs are a source for non-canonical miRNAs [71,91,94,95,96]. Interestingly, components of the canonical miRNA biogenesis pathway such as DICER and DGCR8 are involved in the processing of snoRNA-derived miRNAs and stability of snoRNAs, respectively [95,97,98,99] (Figure 1C). Together with other proteins, DGCR8 can degrade snoRNAs after processing and thereby influences the processing of snoRNA-derived miRNAs [100]. 

Like canonical miRNAs, snoRNA-derived miRNAs are approximately 21 nt long, are bound to AGO1-AGO4 and repress target mRNAs in a miRNA-like fashion [73,95,101]. For instance, the ACA45-derived miRNA binds to the 3′-UTR and inhibits the expression of transcriptional regulator cell division cycle 2-like 6 (CDC2L6) [95]. In addition, snoRNA-derived miR-28 (sno-miR-28) is processed from either SNORD25 or SNORD28 and represses the expression of the P53-stabilizing transcription initiation factor TFIID subunit 9b (TAF9B) [102]. Furthermore, the HBII-336 (SNORD93)-derived miRNA (sdRNA-93) has been shown to exhibit oncogenic features by silencing Pipecolic acid and Sarcosine oxidase (PIPOX) in triple-negative breast cancer (TNBC) and Her2^+^ luminal breast cancer [71,73]. Increased expression of sdRNA-93 in breast cancer enhances the proliferation of tumor cells, contributes to tumor cell invasion, and is associated with aggressiveness of the malignancy. Together, these findings indicate that some snoRNAs are sources of functional non-canonical miRNAs. 

### 2.4. tRNA-Derived miRNAs

A very different source of non-canonical miRNAs are transfer RNAs (tRNAs) [103]. The clover-leaf structure of tRNAs is a substrate for DICER or Angiogenin (ANG), which cleaves the tRNA stem into tRNA-derived RNA (tDR) fragments [104,105,106]. Additionally, recent data indicate that some tRNA fragments are loaded in AGO proteins and regulate gene expression similar to miRNAs [104,107] (Figure 1D). For example, CU1276 is a tDR fragment that is highly expressed in germinal center B-cells and downregulated in diffuse large B-cell lymphoma. Similar to canonical miRNAs, CU1276 represses the expression of replication protein A1 (RPA1), an activity that is crucial for DNA-damage repair, thereby inhibiting the outgrowth of Burkitt’s lymphoma cells [77]. 

Another putative source for tRNA-derived miRNAs are 5′-tRNA stress-induced fragments (tiRNAs). The tRNAVal tiRNA targets Frizzled homolog 3 (*FZD3*) mRNA in a seed-dependent manner in human breast cancer cells [72]. Downregulation of FZD3 inhibits the wingless-type MMTV integration site family (Wnt)/β-catenin pathway and thereby inhibits breast cancer progression and invasion, indicating potential tumor suppressor activities of tiRNAVal [72]. Although these data strongly suggest miRNA-like functions of tiRNAs, it is not known whether tiRNAs are loaded into RISC. 

The Lupus autoantigen (LA) plays a major role in the inhibition of DICER-mediated cleavage of tDR fragments. LA stabilizes POL III transcripts and facilitates the folding of RNAs [108]. Studies in human embryonic kidney (HEK) cells show that LA deficiency results in enhanced tDR fragment-cleavage by DICER and loading of tDR-fragments into AGO [104] (Figure 1D). An exception to this rule is the pre-tRNAIle, which has a low affinity for LA. Instead, pre-tRNAIle is bound by XPO-5 and subsequently translocated to the cytoplasm, where it is cleaved by DICER, processed into miR-1983 and loaded into AGO2 [104]. Subsequently, miR-1983 represses the expression of regulating synaptic membrane exocytosis 2 (RIMS2), BTB domain and CNC homolog (BACH1), and BACH2, which are validated miR-1983 targets. 

Strikingly, upon overexpression of tRNAs, 3′ tDR-fragments are loaded in RISC in a process that is independent from DROSHA, XPO-5, and DICER [109]. These 3′ tDR-fragments have been shown to destabilize their target transcripts in a seed- and RISC-dependent fashion [109]. The factors involved in the biogenesis of these 3′ tDR-fragments remain elusive. This mechanism may be important in human cancers where tRNAs are frequently overexpressed [110]. 

Together, these data show that miRNAs are processed from unexpected non-coding RNAs, by mechanisms that may be cell type- and cellular state-dependent. AGO-CLIP experiments with various cell types under different conditions such as oncogenic stress, DNA damage, and oxidative stress, may allow for the identification of novel miRNA sources.

## 3. Mechanisms of miRNA Nuclear Localization 

In contrast to typical miRNA functions in the cytoplasm, some miRNAs are localized in the nucleus [46,111]. The first observed nuclear miRNA is miR-21, of which 20% of the total amount is localized in the nucleus of HeLa cells [46]. Later, by using in situ hybridization, Politz et al. found that miR-206 co-localizes with 28S rRNA in the nucleolus of rat myogenic cells [112]. With live cell imaging technology, Földes-Papp et al. showed that miR-122 is actively transported from the cytoplasm to the nucleus of human liver cells [113]. More intriguing observations of nuclear miRNAs followed and raised questions about nuclear miRNA processing, trafficking, and functions.

Recent data suggest that there are three mechanisms behind the nuclear localization of miRNAs which may not be mutually exclusive including: (1) nuclear localization signal sequences within a subset of miRNAs, (2) full processing of a subset of miRNAs in the nucleus, and (3) continuous shuttling of miRNAs of which a subset of miRNAs enriches in the nucleus due to interaction with their targets.

### 3.1. Sequence-Dependent Nuclear Localization

**Figure 2:**
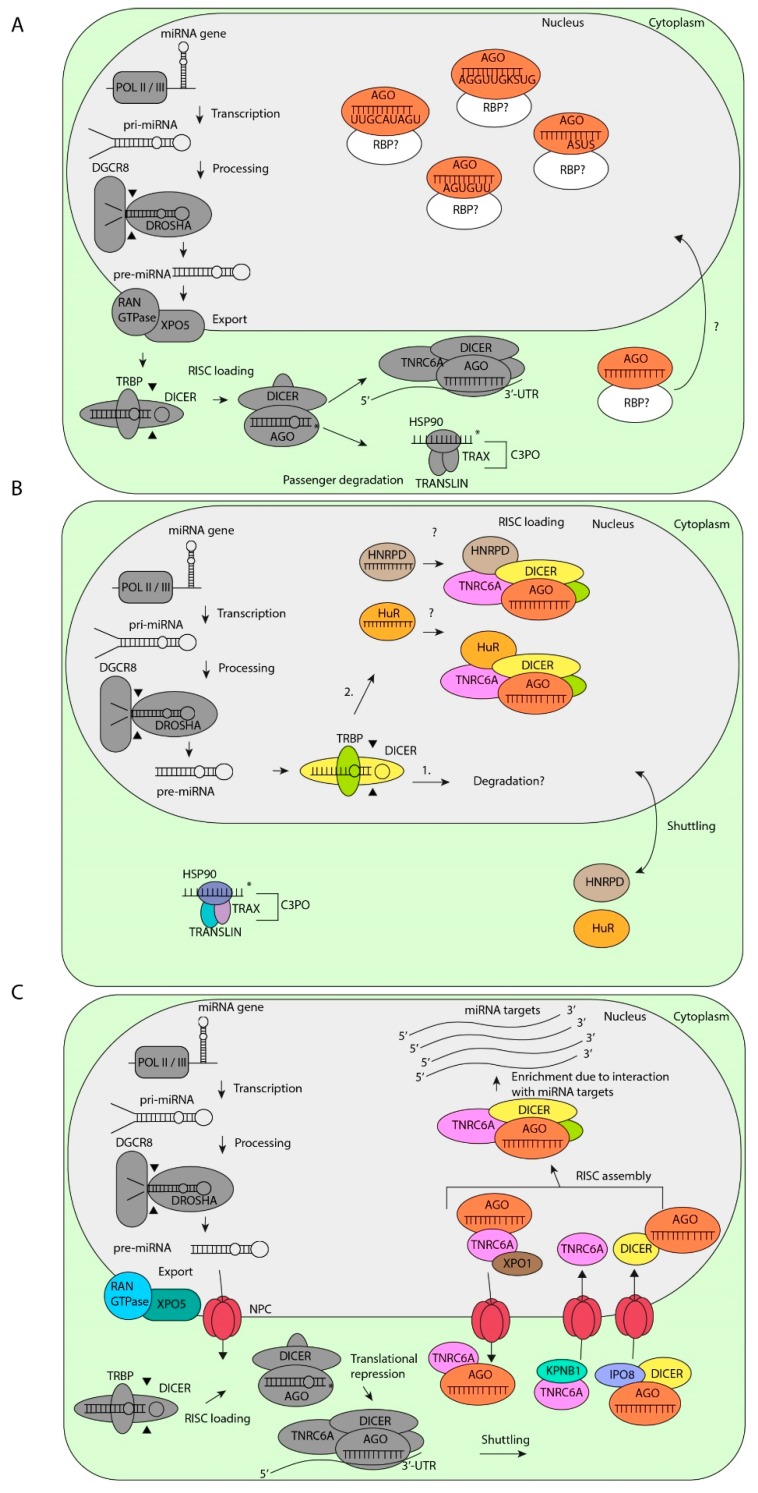
Nuclear localization mechanisms of miRNAs. (**A**) After canonical processing of pri-miRNAs, miRNAs containing specific motifs are transported back into the nucleus by still unknown RNA-binding proteins (RBPs) and mechanisms. (**B**) After processing by DROSHA/DGCR8, pre-miRNAs will be processed by nuclear DICER/TRBP for (1) degradation or (2) RISC loading in the nucleus by heterogeneous nuclear ribonucleoprotein D (HNRPD) or human antigen R (HuR), which both shuttle between the cytoplasm and the nucleus by an unknown mechanism. (**C**) After processing by DROSHA/DGCR8, miRNAs bind to XPO-5 and will be exported trough the nuclear pore complex (NPC). Mature miRNAs bound by AGO2 will be imported back into the nucleus by DICER and Importin 8 (IPO8). TNRC6A will be imported into the nucleus by Karyopherin β1 (KPNB1). Subsequently, RISC will be assembled in the nucleus and will interact with miRNA targets, which allows for miRNA enrichment in the nucleus. Ultimately, AGO2 and TNRC6A will be exported back to the nucleus by XPO1.

An extensively studied nuclear miRNA is miR-29b. This miRNA is a member of the miR-29 family and differs from its family members, miR-29a and miR-29c, by an uridine residue at nucleotide 10 and an AGUGUU-motif at the 3′ end [114]. This hexanucleotide sequence, but not the uridine residue, is responsible for the nuclear localization of miR-29b in HeLa, nasopharyngeal cancer, NIH 3T3 cells and mouse endothelial yolk sac cells [111,115,116,117,118]. This AGUGUU-motif is present in many miRNAs [111,119] (Table 1). Moreover, one-third of nuclear miRNAs contain the consensus ASUS sequence, where S is a cytosine or a guanidine [120]. In addition, miRNAs with 5′–UUGCAUAGU–3′ and 5′–AGGUUGKSUG–3′ motifs, where K is a uridine or a guanine, are also translocated to the nucleus of murine epithelial yolk sac cells [117]. These motifs are mainly present in the 5p-arm of let-7 family members [117]. Thus nuclear localization of miRNAs is mediated by different motifs, in a mechanism that may be controlled by specific RNA-binding proteins (RBPs). However, the molecular pathways and RBPs that specifically bind to these motifs and translocate miRNAs to the nucleus remain to be identified (Figure 2A).

Several studies show that miRNAs, including, e.g., miR-21, miR-29a, which do not contain known nuclear localization sequences, are also localized in the nucleus of some cell types [121,122]. Moreover, deep sequencing and microarray profiling studies even revealed that most miRNAs are imported into the nucleus of nasopharyngeal and colon cancer cells [115,122]. These data strongly suggest that a general mechanism controls the nuclear localization of miRNAs in some cell types and/or under certain specific conditions.

### 3.2. Nuclear Biogenesis of miRNAs

In the process of canonical miRNA biogenesis, pre-miRNAs are cleaved by DICER/TRBP in the cytoplasm [3]. However, there is strong evidence for DICER activity in the nucleus under specific circumstances [123,124,125,126]. In agreement, by studying the different protein domains of DICER in HeLa cells, Doyle et al. found that the dsRNA binding domain of DICER also functions as a nuclear localization signal (NLS) [126]. 

In addition, several studies have shown that RISC components such as human AGO1-4, TRBP and TNRC6A are also localized in nuclei of mammalian cells [123,127,128,129,130,131,132]. However, this does not necessarily mean that nuclear localized miRNAs are loaded in RISC in the nucleus. Are nuclear processed miRNAs loaded into RISC in the nucleus? The argument against nuclear RISC loading is the fact that proteins important for RISC loading, such as HSP90, TRAX, and TRANSLIN are all exclusively localized in the cytoplasm [123]. These data strongly suggest that nuclear miRNAs are not functional, but are instead degraded after processing by DROSHA and DICER in the nucleus as part of a negative feedback mechanism.

However, this does not rule out a different mechanism for nuclear RISC loading by alternative factors than the canonical RLC and there are some indications for such a mechanism. For example, AU-rich-binding factor 1 (AUF1), also known as heterogeneous nuclear ribonucleoprotein D (HNRPD) has been shown to bind Let-7b by one of two RNA recognition motifs (RRM) and load Let-7b into AGO2 [133]. Interestingly, HNRPD has been found to shuttle between the cytoplasm and nucleus [134] and may be a candidate RBP involved in RISC loading of specific miRNAs in the nucleus. Similarly, the RBP human antigen R (HuR), which is localized in both the nucleus and cytoplasm, binds Let-7b and Let-7i and loads these miRNAs into AGO2 [135]. Although this is still speculative, we hypothesize that processed miRNAs may be loaded into RISC by specific RBPs in the nucleus (Figure 2B). However, this model still needs proper validation. 

### 3.3. Target-Specific miRNA Enrichment in the Nucleus by RISC Shuttling

There is emerging evidence that RISC components continuously shuttle between the nucleus and cytoplasm. Proteins from the Karyopherin family such as Exportin-1 (XPO1), Importin-8 (IPO8), Karyopherin β (KPNB1) and XPO5 mediate the shuttling of proteins that contain a classical nuclear localization signal (NLS) and nuclear export signal (NES), through the nuclear pore complex (NPC) [136,137]. RISC components such as TNRC6A and DICER contain an NLS and translocate from the cytoplasm to the nucleus by binding to IPO8 and KPNB1, respectively [126,129,138]. AGO2 does not contain a classical NLS. However, IPO8 co-localizes with AGO2 in human and murine cells [139,140]. This can only be explained when AGO2 is bound to RISC components such as DICER and is subsequently translocated to the nucleus. However, the direct interaction between AGO2, IPO8, and DICER has not been investigated yet. 

To be transported out of the nucleus, miRNA-bound AGO2 is dependent on TNRC6A, which also contains an NES [129]. TNRC6A contains three known binding sites for AGO2, of which two play a critical role in the export of miRNA-bound AGO2 to the cytoplasm via XPO1 [129,137]. Furthermore, the interaction of XPO1 with RISC is RNA-dependent, indicating that the interaction of miRNA with AGO is a requirement for RISC export from the nucleus to the cytoplasm [141]. 

As AGO2 has no preference for specific miRNA sequences [142,143], all miRNAs are expected to shuttle between the cytoplasm and the nucleus when this mechanism is active in the cell. The question then also arises whether these miRNAs are active in the nucleus. A recent study in embryonal stem cells presents strong evidence that nuclear miRNAs bind to 3′-UTRs, introns, protein-coding sequences and 5′-UTRs of pre-mRNAs in a seed-dependent manner [132]. 

When all miRNAs actively shuttle between the cytoplasm and the nucleus, why are some miRNAs still enriched in the nucleus? The enrichment can be explained by a model in which only the miRNAs that are bound to nuclear targets are found predominantly in the nucleus (Figure 2C). The evidence for this model came from experiments from Pitchiaya et al., in which micro-injection of RNA into the nucleus results in nuclear enrichment of only those miRNAs that have seed-matches in the nuclear injected RNAs [121]. Thus, in this model, miRNA loaded RISC shuttles continuously between the cytoplasm and the nucleus and only the miRNAs that are bound to their targets are enriched in the nucleus. In conclusion, the shuttling of miRNAs between the cytoplasm and the nucleus is controlled by different mechanisms, which are largely dependent on the cell type and cellular state. The non-canonical activities of miRNAs in the nucleus are discussed in the section below.

## 4. MiRNA-Mediated Transcriptional Regulation

**Figure 3:**
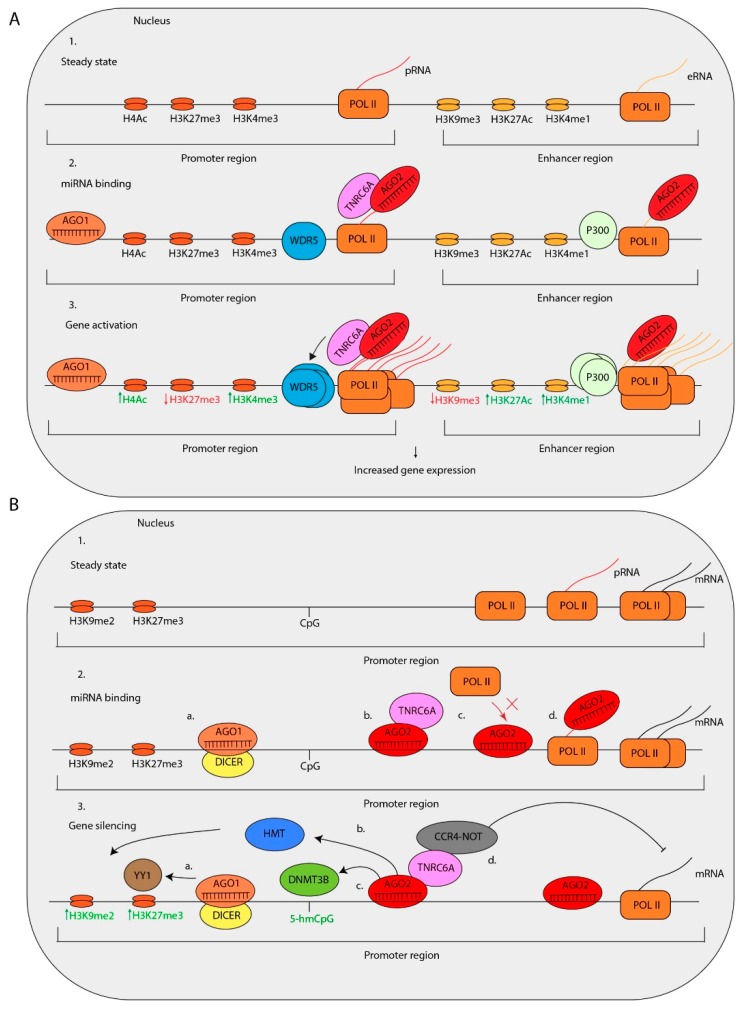
MiRNA-mediated transcriptional regulation is shown in three steps. (**A**) (1) In steady state, POL II will transcribe promoter RNAs (pRNAs) in promoter regions and enhancer RNAs (eRNAs) in enhancer regions. (2) MiRNA-loaded AGO1 can interact with promoter DNA. MiRNA-loaded AGO2 can bind to pRNAs, which recruits WD repeat-containing protein 5 (WDR5) to the promoter, or to eRNAs which recruits P300 to the enhancer region. (3) The interaction of AGO1 with promoter regions causes POL II and histone H3 Lysine 4 trimethylation (H3K4me3) enrichment on the promoter. The interaction of AGO2 with pRNAs causes WDR5 and POL II enrichment on promoters, which leads to a decrease in histone H3 Lysine 27 trimethylation (H3K27me3) and increase in histone H4 acetylation (H4Ac) levels, accompanied by an increase in pRNA transcription. The interaction of AGO2 with eRNAs causes enrichment of P300 and POL II on enhancer regions, an increase of local histone H3 Lysine 27 acetylaction (H3K27Ac) and histone H3 Lysine 4 monomethylation (H3K4me1) levels, a decrease of H3K27me3 levels and an increase of eRNA expression. These processes ultimately lead to increased gene expression. (**B**) MiRNAs are involved in transcriptional gene silencing, which are depicted in three steps. (1) In steady state, POL II will be enriched on promoter regions, resulting in gene transcription. (2a) MiRNA-loaded AGO1/DICER complex interacts with promoter regions. (2b) MiRNA-loaded AGO2/TNRC6A-complex interacts with promoter regions. (2c) MiRNA-loaded AGO2 interacts with the promoter region, and prevents POL II binding to the promoter. (2d) MiRNA-loaded AGO2 binds to pRNAs. (3a) MiRNA-loaded AGO1/DICER complex interacts with the promoter region and recruits Yin Yang 1 (YY1), which results in increased H3K27me3 levels. (3b) MiRNA-loaded AGO2 recruits histone methyltransferases (HMTs) to increase H3K9me2 and H3K27me3 levels. (3c) MiRNA-loaded AGO2 recruits DNA-methyltransferase 3B (DNMT3B) to methylate CpGs in the genome and silences gene expression. (3d) MiRNA-loaded AGO2/TNRC6A complex recruits the CCR4-NOT complex to degrade POL II-derived transcripts. These processes will ultimately lead to transcriptional gene silencing.

Here, we discuss whether miRNAs are active in the nucleus. Increasing evidence shows that this is true. For instance, several miRNAs that are prominently localized in the nucleus have been associated with transcriptional regulation [144]. Many miRNA-binding sites in gene promoters in sense and antisense orientations have been predicted by in silico analyses [145]. Also, functional experiments in mammalian cells provide strong evidence that miRNAs regulate the transcription of target genes by binding to reverse complementary sequences in promoter regions [117,145]. Below, we review two functions of nuclear miRNAs that have been identified: first, transcriptional gene activation (TGA) (Figure 3A) and second, transcriptional gene silencing (TGS) (Figure 3B).

### 4.1. Transcriptional Gene Activation

Several miRNAs have been associated with increased expression of target genes. For example, transfection of miR-373 in prostate cancer cells induced the expression of E-cadherin and cold shock domain-containing protein C2 (CSDC2) by interacting with reverse complementary sequences at their transcriptional start sites [146]. Additionally, three different miRNAs, miR-744-5p and miR-466d-3p, enhance the expression of Cyclin B1 (*CCNB1*), a gene that lacks miRNA binding sites of these miRNAs in the 3′-UTR. Instead, these miRNAs match almost completely with a sequence motif located in the promoter [147]. The induction of *CCNB1* expression is dependent on miRNA biogenesis factors such as DROSHA, DICER, AGO1, and AGO2 [147]. Furthermore, the transcriptional upregulation is accompanied by the enrichment of AGO1, and POL II and an increase in histone H3 Lysine 4 trimethylation (H3K4me3) near the transcriptional start site of *CCNB1* [147]. 

Another example is miR-205, which interacts with reverse complementary binding sites in the promoter of *IL35* and *IL24*. Ectopic overexpression of miR-205 increases the expression of IL-35 and IL-24 accompanied by enrichment of POL II and histone marks associated with active transcription [78]. Recent studies on miRNA-mediated TGA indicate that this is cell type-specific. For instance, the expression of miR-1236-3p, miR-370-5p, and miR-3619-5p, all binding to matching sites in the *P21* promoter, has been associated with an increased *P21* expression in human endometrial cancer, pancreatic cancer, and lung carcinoma cell lines, but not in liver cancer cells [148,149]. By performing biotinylated RNA-affinity pull-down experiments, it has been shown that miR-3619-5p directly interacts with the *P21* promoter. This interaction induces the expression of P21, thereby inhibiting the proliferation of prostate cancer cells [149]. 

Matsui et al. present evidence that miR-589 induces the expression of Cyclooxygenase-2 (*COX-2*) and Phospholipase A2 (*PLA2G4A*) in A549 lung cancer cells via a different mechanism [76]. The human *COX-2* promoter contains two miR-589 seed matches [76]. The promoter-associated RNAs (pRNAs) of *COX-2* are transcribed in a bi-directional fashion at low levels [76]. MiR-589 in complex with AGO2 and TNRC6A interacts *COX-2* pRNAs and enhances its expression by an unknown mechanism. Correspondingly, this interaction enhances the expression of *COX-2* [76]. The miR-589 binding to the *COX-2* pRNA induces chromatin marks associated with gene activation, such as H3K4me3 and histone H4 acetlyation (H4Ac) [76]. Interestingly, upon gene activation and RISC binding, the WD repeat-containing protein 5 (WDR5), a factor which recruits various histone methyltransferases (HMTs), and associated with gene activation, is recruited to the promoter of the *COX-2* locus [76] [150,151]. Chromosome conformation capture (3C) analysis indicated that the interaction of miR-589 with the *COX-2* pRNA induces a physical interaction between the *COX-2* and *PLA2G4A* gene promoters and mediates a simultaneous induction of *COX-2* and *PLA2G4A* expression [76]. 

Interestingly, a different study provides evidence that over 300 miRNA genes in the human genome are located in enhancer regions of protein-coding genes. Remarkably, the expression of these miRNAs is positively correlated with the expression of the neighboring protein-encoding genes [152]. Moreover, the expression of the miRNA is in some cases critical for enhancer activity. For example, miR-24-1 is located in an enhancer region. Ectopic expression of miR-24-1 induces the expression of the flanking genes encoding Fructose-1,6-biphosphatase 1 (FBP1) and Fanconi anemia complementation group C (FANCC). This miRNA enhancer activity is absent in cells that are deficient for miR-24-1 [152]. Surprisingly, miR-24-1-mediated expression of FBP1 is accompanied by POL II, P300 and histone H3 Lysine 27 acetylation (H3K27Ac) enrichment and an increase in enhancer RNA (eRNA) expression. Interestingly, miR-24-1 is involved in the upregulation of FBP1 and FANCC in an AGO2- and miR-24-1 seed-dependent manner [152]. These data suggest that miR-24-1 regulates the expression of flanking genes by the activation of eRNA expression. In conclusion, these data reveal a complex network in which nuclear miRNAs mediate transcriptional gene activation by various mechanisms.

### 4.2. Transcriptional Gene Silencing 

Nuclear localized miRNAs have also been associated with gene silencing. In addition to canonical miRNA functions, Miao et al. have shown evidence that miR-552 has non-canonical functions in the nucleus. This study indicated that miR-552 inhibits the expression of human cytochrome P450 2E1 (*CYP2E1*) in two independent manners [153]. First, the 3′ UTR of the *CYP2E1* mRNA contains an imperfect miR-552 binding site with one mismatch in the miR-552 seed. The investigators confirmed that miR-552 inhibits the translation of *CYP2E1* mRNA [153]. Second, the 3′-end of miR-552 is perfectly complementary to a region in the promoter of *CYP2E1*. The *CYP2E1* promoter folds in a DNA cruciform structure, of which the loop contains the miR-552 binding site. A cruciform structure in gene promoters is often involved in transcriptional regulation [154]. Both the *CYP2E1* promoter and miR-552 form a DNA:RNA hybrid, which regulates *CYP2E1* expression [153]. An increase in miR-552 expression causes a decrease in POL II and transcription factor binding at the *CYP2E1* promoter, thereby silencing *CYP2E1* transcription [153]. However, whether this is caused by the recruitment of downstream proteins or simply the occupation of RISC on the promoter region and thereby preventing POL II binding is unknown. 

In a different study, Let-7f-loaded AGO2 was shown to repress genomic DNA targets by interacting with the retinoblastoma protein (Rb) in senescent human breast cancer cells. In these cells AGO2 and Rb are co-localized at genes that are controlled by E2F [155]. Upon induction of senescence, AGO2 and Rb cause a Let7f-dependent decreased expression of E2F target genes. This interaction also recruits histone deacetylases (HDACs) and HMTs and as a result increases inactive chromatin marks histone H3 Lysine 9 dimethylation H3K9me2 and histone H3 Lysine 27 trimethylation H3K27me3 at the targeted locus [155]. Whether AGO2- and Let-7f-dependent gene silencing occurs by miRNA:DNA or miRNA:pRNA interactions at the promoters of target genes is unknown [155]. However, several other studies have shown evidence for miRNA-mediated recruitment of HDACs, DNA-methyltransferases (DNMTs), and HMTs via interaction with pRNA, suggesting that miRNA:pRNA interactions are more common [156,157,158,159]. Another example of miRNA-mediated DNA methylation is described for miR-133a. The promoter of *Dnmt3b* contains a miR-133a binding site in pluripotent stem cells [144]. The interaction of miR-133a with the promoter of *Dnmt3b* recruits DNMT3B via direct binding to AGO2. This subsequently results in increased H3K27me3 levels, methylation of the *Dnmt3b*-promoter and inactivation of DNMT3B expression. In addition, miR-223 is involved in the silencing of nuclear factor I-A (NFI-A) and thereby affects granulopoiesis [75]. During granulopoiesis, miR-223 is bound to sites within the promoter of NFI-A. The miR-223-RISC recruits Polycomb group complex (PcG) to the promoter of NFI-A by interacting with Yin Yang 1 (YY1) which is an important Polycomb group complex (PcG) subunit. The PcG complexes are involved in the recruitment of histone-modifying enzymes to mediate differentiation processes [160]. The miR-223-mediated silencing of NFI-A is an important step in the induction of granulocytic differentiation [75]. 

Nuclear RISC may contain other effector protein complexes, such as the CCR4-NOT complex. Recent studies indicate that the AGO1/2 interacting protein TNRC6A functions as a scaffold protein to recruit CCR4-NOT complex to specific loci in the genome and mediate TGS [129,161]. A recent study shows that the CCR4-NOT complex is critical for the degradation of targets of nuclear-localized miRNAs in stem cells [132]. The depletion of the CCR4-NOT factors, including NOT1 and NOT7, causes an increased stability of pre-mRNAs containing nuclear miRNA target sites [132]. These data indicate that these factors are key players in nuclear miRNA-mediated pre-mRNA transcript degradation which is important for the fine-tuning of gene expression.

Interestingly, some nuclear miRNAs silence gene expression in a very different way. For instance, the circular antisense cerebellar degeneration-related protein 1 (*CDR1*) transcript (AS-CDR1) stabilizes *CDR1* transcripts [162]. Therefore, the expression of AS-CDR1 is positively correlated with the expression of CDR1 mRNA. The expression of AS-CDR1 is controlled by miR-671 in an AGO2-dependent manner [162]. Cleavage of AS-CDR1 is dependent on a near-perfect binding site of miR-671 containing a centered mismatch of 2 nt at position 14 and 15 [162]. As expected, cloning 5′-rapid amplification of cDNA ends (RACE) cleavage products of AS-CDR1 determined that the AGO2 cleavage site is located at position 9–11 nt of miR-671 [162]. In conclusion, miRISC induces TGS by interacting with chromosomal DNA, antisense transcripts or pRNAs and subsequently recruits epigenetic modifiers or cleave pRNA and antisense transcripts to induce silencing of target genes.

## 5. Conclusions

It is becoming evident that miRNAs do not only originate from miRNA genes, but are also processed from other non-coding RNAs, such as snoRNAs and tRNAs. As current miRNA research mainly focuses on the canonical aspects of miRNAs, this causes a potential underestimation of the effects of miRNAs on transcriptional gene regulation including gene transcription and gene silencing. Recent experimental observations indicate that the nuclear localization of miRNAs is not an exception to the rule. However, further research is required to understand the different mechanisms involved in the shuttling of miRNAs between the cytoplasm and nucleus. The identification of novel RNA-binding proteins involved in these processes may lead to a better understanding of nuclear miRNA biology. Together, these findings add novel levels of complexity to miRNA functions. Further research is required to shed more light on the understanding of the complex regulatory functions of miRNAs.

## Figures and Tables

**Table 1:** Nuclear localization sequences in miRNAs.

Sequence	miRNAs	Localization	Reference
**UUGCAUAGU**	let-7e-5p, let-7a-5p, let-7c-5p, let-7d-5p, let-7f-5p	Nucleus	[117]
AGGUUGKSUG *	let-7b-5p, let-7e-5p, let-7a-5p, let-7c-5p, let-7d-5p, let-7f-5p	Nucleus	[117]
**AGUGUU**	miR-29b-3p, miR-199b-5p, miR-199a-5p, miR-200c-5p, miR-141-5p	Nucleus	[111,115,116,119,121,122]
**ACUGUU**	miR-452-5p, miR-203b-3p	Nucleus	[115]
**AGUGAU**	miR-7-5p, miR-573, miR-338-3p	Nucleus	[115]
**AGUGUA**	miR-449a, miR-222-5p, miR-532-5p, miR-34c-5p	Nucleus	[115,122]
ASUS *	miR-30b-5p, miR-30c-5p, miR-19a-3p, miR-374a-5p, miR-374b-5p, miR-590-5p, miR-193b-3p	Nucleus	[120]

* S is a cytosine or guanine, K a guanine or thymidine.
